# Supplementary material for: Content-rich biological network constructed by mining PubMed abstracts
Source: BMC Bioinformatics. 2004 Oct 8;5:147. doi: 10.1186/1471-2105-5-147 (PMC528731; doi:10.1186/1471-2105-5-147)
Supplement: Additional File 2 — The original results of the above study (non-essential files are deleted to keep the file size under the limit set by BMC bioinformatics). [file 1471-2105-5-147-S2.bz2 › chilibotAdditionalFile2/dip05/547ID8872471E10900/html/SCA1_GAPD.html]

 


 **SCA1** and **GAPD** 
  
Found 2 abstracts in PubMed, retrieved 2.  
 

 What does Google say? 
 PDF only 
| .edu only 

---

**Interactive relationship** (e.g. stimulation, inhibition, etc)

**Neutral relationship**- Spinocerebellar ataxia type 1  [ **SCA1** ]  and spinobulbar muscular atrophy gene products interact with glyceraldehyde 3 phosphate dehydrogenase  [ **GAPD** ] .  Ref: 8872471 Hum Mol Genet, 1996
- Physical interaction between GAPDH  [ **GAPD** ]  and ataxin 1  [ **SCA1** ]  was also demonstrated in vitro.  Ref: 8872471 Hum Mol Genet, 1996

**Non-interactive relationship** (e.g. studied together, co-existance, homology, etc.)

- The binding of both ataxin 1  [ **SCA1** ]  and the androgen receptor to GAPDH  [ **GAPD** ]  does not vary with the length of the polyglutamine tract.  Ref: 8872471 Hum Mol Genet, 1996
- PATIENTS AND METHODS Activity of GAPDH  [ **GAPD** ]  was measured in morphologically affected and unaffected brain areas of patients with 4 different CAG repeat disorders Huntington disease, spinocerebellar ataxia 1  [ **SCA1** ]   **SCA1** , SCA2, and SCA3 Machado Joseph disease, in brains of patients with Friedreich ataxia a GAA repeat disorder and Alzheimer disease, and in brains of matched control subjects.  Ref: 9779656 Arch Neurol, 1998
- In this study we used ataxin 1  [ **SCA1** ] , the  **SCA1**  gene product, as a bait in the yeast two hybrid system and identified the glycolytic enzyme glyceraldehyde 3 phosphate dehydrogenase  [ **GAPD** ]  as an ataxin 1  [ **SCA1** ]  interacting protein.  Ref: 8872471 Hum Mol Genet, 1996
